# Iron metabolism disorder promotes postovulatory oocyte aging by inducing oxidative stress damage

**DOI:** 10.1093/lifemedi/lnaf032

**Published:** 2025-09-30

**Authors:** Ziqi Huang, Bicheng Wang, Ying Tian, Xiangning Xu, Jiaqi Zhang, Shuo Lou, Jingyi Kang, Ningning Zhang, Ke Song, Jingyu Li, Jing Weng, Yuanjing Liang, Xiaokui Yang, Wei Ma

**Affiliations:** Department of Histology and Embryology, School of Basic Medical Sciences, Capital Medical University, Beijing 100069, China; Department of Histology and Embryology, School of Basic Medical Sciences, Capital Medical University, Beijing 100069, China; Department of Histology and Embryology, School of Basic Medical Sciences, Capital Medical University, Beijing 100069, China; Department of Histology and Embryology, School of Basic Medical Sciences, Capital Medical University, Beijing 100069, China; Department of Histology and Embryology, School of Basic Medical Sciences, Capital Medical University, Beijing 100069, China; Department of Histology and Embryology, School of Basic Medical Sciences, Capital Medical University, Beijing 100069, China; Department of Histology and Embryology, School of Basic Medical Sciences, Capital Medical University, Beijing 100069, China; Department of Histology and Embryology, School of Basic Medical Sciences, Capital Medical University, Beijing 100069, China; Department of Human Reproductive Medicine, Beijing Obstetrics and Gynecology Hospital, Capital Medical University, Beijing 100020, China; Department of Human Reproductive Medicine, Beijing Obstetrics and Gynecology Hospital, Capital Medical University, Beijing 100020, China; Department of Histology and Embryology, School of Basic Medical Sciences, Capital Medical University, Beijing 100069, China; Department of Histology and Embryology, School of Basic Medical Sciences, Capital Medical University, Beijing 100069, China; Department of Human Reproductive Medicine, Beijing Obstetrics and Gynecology Hospital, Capital Medical University, Beijing 100020, China; Department of Histology and Embryology, School of Basic Medical Sciences, Capital Medical University, Beijing 100069, China

**Keywords:** oocyte, postovulatory aging, iron, ferritinophagy, quality deterioration

## Abstract

Reactive oxygen species (ROS) involve in oocyte postovulatory aging, yet the mechanism of ROS accumulation is not fully understood. We explored iron metabolic status and its relationship with ROS in mouse oocytes during postovulatory aging *in vivo*. We found that heme oxygenase 1 (HO-1) expression was increased in oviduct and iron metabolism was disordered in oocytes with time post-ovulation. The aging oocytes were manifested with high iron content and disturbed expressions of iron metabolic proteins, including ferritin heavy chain (FHC), mitochondrial ferritin (FtMT), divalent metal transporter 1 (DMT1), ferroportin1 (FPN1), iron regulatory protein 2 (IRP2) and transferrin receptor 1 (TFR1), along with increased cytosolic free Fe^2+^, lipid peroxidation, DNA damage, mitochondrial and lysosomal abnormality, and defects in spindle and chromosome alignment. These *in vivo* aging cells contained stable glutathione peroxidase 4 (GPX4) and 4-hydroxynonenal (4-HNE), unexpectedly, but had more iron and degenerative changes than their *in vitro* counterparts. The intraperitoneal deferoxamine (DFO) could alleviate all these changes and improve the fertilization competence and preimplantation development. Similarly, the HO-1 inhibitor Zinc Protoporphyrin (ZnPP) also could do this. Together, the iron homeostasis disturbance participates in ROS accumulation and degenerative changes in postovulatory aging oocytes, which can be alleviated by iron chelating.

## Introduction

Postovulatory oocyte aging refers to the time-dependent degradation of ovulated oocytes, leading to fertilization failure and abnormal embryonic development. The degenerative changes associated with this process are closely linked to the excessive ROS accumulation. ROS can originate as byproducts of oxidative phosphorylation within the mitochondrial electron transport chain (ETC) [1], and also arise from iron-mediated Fenton reactions, where transition metal ions (Fe^2+^) catalyze the decomposition of hydrogen peroxide (H_2_O_2_) to form the hydroxyl radical (•OH), one of the most harmful free radical molecules [2]. Maintenance of cellular iron homeostasis relies on the coordinated actions of iron transporters, carriers, regulatory proteins, and storage proteins [3]. When this balance is disrupted, cytosolic free Fe^2+^ levels increase, thereby expanding the intracellular labile iron pool (LIP). This elevated LIP enhances Fenton reactions and hydroxyl radical (•OH) generation, which typically leads to enhanced lipid peroxidation and cellular damage, ultimately triggering cell ferroptosis [4].

Here, we demonstrate that iron homeostasis is progressively disrupted in oocytes following ovulation. With post-ovulatory aging, oocytes exhibit elevated cytosolic Fe^2+^ levels, increased ferritinophagic and mitophagic flux, and concomitant oxidative stress damage. *In vivo* iron chelation effectively reduces cytosolic Fe^2+^ accumulation, dampens the hyperactivity of ferritinophagy and mitophagy, and alleviates lipid peroxidation damages. Critically, this intervention also improves oocyte fertilization competence and preimplantation embryo development. These findings reveal a previously unrecognized link between oocyte aging and iron dysregulation, suggesting novel therapeutic avenues for reproductive pathologies.

## Results

### Oviductal HO-1 increase and oocyte iron metabolism disorder with time post-ovulation

**Figure 1.**
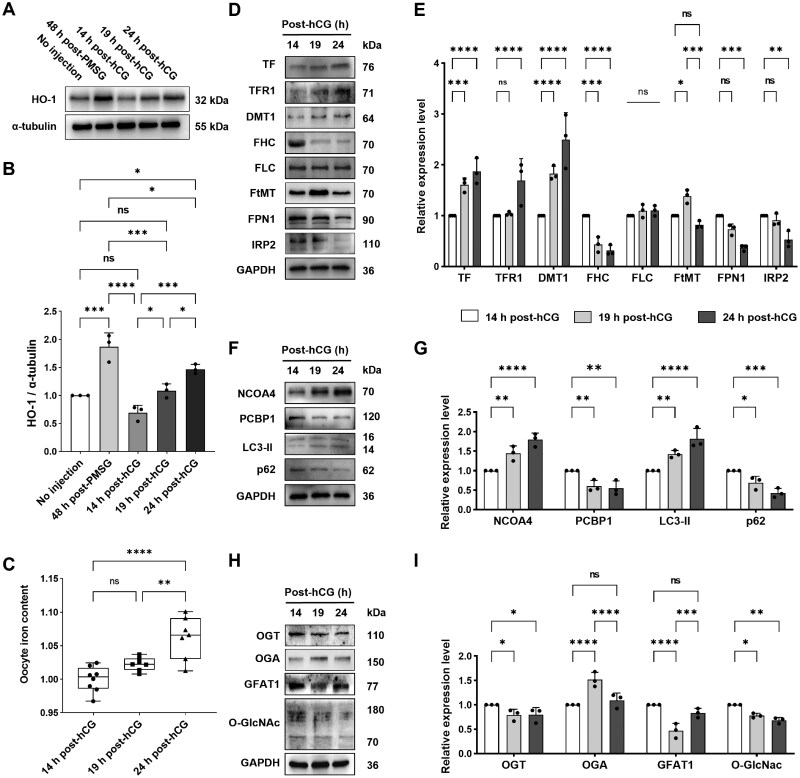
Changes in HO-1 expression in oviducts and iron metabolism regulation in mouse oocytes with time after ovulation. (A) Representative images of Western blot analysis about HO-1 expression in mouse oviduct tissues before and after ovulation along PMSG/hCG superovulation protocol. (B) Statistical analysis of blot gray scale value of HO-1 in different groups. Oviducts from 15 mice were used in this experiment. (C) The change of iron content in oocytes during postovulatory aging. ICP-MS was conducted on oocytes collected at 14 h, 19 h and 24 h after hCG administration. (D) Representative images of immune blots of TF, TFR1, DMT1, FHC, FLC, FtMT, FPN1 and IRP2 in mouse oocytes. (E) Statistical analysis about the blot gray scale value of TF, TFR1, DMT1, FHC, FLC, FtMT, FPN1 and IRP2 in different groups. (F) Representative images of immune blots of NCOA4, PCBP1, LC3-II and p62 in mouse oocytes. (G) Statistical analysis of blot gray value of NCOA4, PCBP1, LC3-II and p62 in different groups. (H) Representative images of immune blots of OGT, OGA, GFAT1 and *O*-GlcNAc in mouse oocytes. (I) Statistical analysis of gray scale value difference of OGT, OGA, GFAT1 and *O*-GlcNAc in different groups. The results were presented as the mean percentage (mean ± SEM) of at least three independent experiments. ns, *P* > 0.05, *P < 0.05, **P < 0.01, ***P < 0.001, ****P < 0.0001. Figure 1A–C were tested by ordinary one-way ANOVA analysis, and Fig. 1D–I were tested by ordinary two-way ANOVA analysis.

Heme oxygenase-1 (HO-1) catalyzes the generation of Fe^2+^ from heme degradation [5]. We first characterized the expression pattern of HO-1 in mouse oviduct tissue along the superovulation schedule by Western blot. As showed in Fig. 1A, oviductal HO-1 expression remained low before PMSG injection, sharply increased at 48 h post-PMSG, and subsequently declined to baseline levels by 14 h post-hCG (approximating pre-injection levels). Following this, HO-1 levels exhibited a sustained upward trend, marked by a significant increase at 19 h post-hCG and a further rise to an even higher peak at 24 h post-hCG (Fig. 1A and 1B). According to our superovulation protocol, oocytes are expected to be ovulated and located in the oviductal ampulla by 14 h post-hCG. Therefore, the subsequent upregulation of HO-1 suggests enhanced heme catabolism in the oviduct, potentially creating an iron-rich microenvironment around the oocytes. Consistent with this hypothesis, ICP-MS analysis revealed an increasing trend in absolute oocyte iron content over time post-hCG. Specifically, intra-oocyte iron levels were significantly elevated at 24 h compared to 14 h and 19 h post-hCG (Fig. 1C).

The observed changes in iron content correlated with the expression patterns of iron metabolism regulatory proteins in aging oocytes. Western blot analysis revealed that the levels of iron-importing proteins, transferrin (TF) and transferrin receptor 1 (TFR1) [3], were elevated, whereas the level of the iron-exporting protein ferroportin 1 (FPN1) [5] was reduced at 19 h and 24 h post-hCG compared to 14 h post-hCG (Fig. 1D lane 1, 2, 7 and 1E). Concurrently, the content of the metal-ion transporter 1 (DMT1) continuously increased after 14 h post-hCG (Fig. 1D lane 3 and 1E). These findings indicate that, with increasing time post-ovulation, oocytes exhibit enhanced iron uptake but diminished iron efflux, coupled with active ferrous iron transport. Regarding iron storage, the ferritin heavy chain (FHC) [6] was significantly decreased, while the ferritin light chain (FLC) [7] remained unchanged at 19 h and 24 h post-hCG (Fig. 1D lane 4, 5 and 1E). Interestingly, mitochondrial ferritin (FtMT) [3] initially increased at 19 h but subsequently decreased at 24 h relative to 14 h post-hCG (Fig. 1D lane 6 and 1E). Collectively, these data suggest a decline in overall iron storage within oocytes over time, potentially leading to increased release of ferrous iron into the cytosol. Unexpectedly, the protein level of iron regulatory protein 2 (IRP2) [5] exhibited a continuous decline after 14 h post-hCG (Fig. 1D lane 8 and 1E). This decreasing trend in IRP2 is consistent with the potential increase in iron content within aging oocytes, however, it is at odds with the upregulation of TFR1 and DMT1, as well as the downward trend for FHC and FtMT expression.

We observed a progressive increase in the protein level of nuclear receptor coactivator 4 (NCOA4), a mediator of ferritinophagy [4], in oocytes from 14 h to 24 h post-hCG (Fig. 1F lane 1 and 1G). In contrast, the expression of poly (rC)-binding protein 1 (PCBP1), a cytoplasmic iron chaperone and negative regulator of ferritinophagy [4], exhibited a time-dependent decrease (Fig. 1F lane 2 and 1G). Consistent with these changes in NCOA4 and PCBP1, the autophagosome marker LC3-II increased, whereas the selective autophagy substrate protein p62 decreased, from 14 h to 24 h post-hCG. (Fig. 1F lane 3, 4 and 1G). Collectively, these changes indicate a hyperactive ferritinophagy process in aging oocytes, potentially linked to ferritin degradation and subsequent Fe^2+^ release into the cytosol. Given that ferritin proteins are more susceptible to autophagic degradation upon loss of O-linked-N-acetylglucosaminylation (*O*-GlcNAcylation) modification [8], we observed a steady decline in *O*-GlcNAc transferase (OGT) activity following ovulation. Conversely, *O*-GlcNAcase (OGA) activity significantly increased at 19 h post-hCG treatment and remained elevated at 24 h post-hCG, albeit not reaching statistical significance (Fig. 1H lane 1–2 and 1I). Furthermore, the level of glutamine-fructose-6-phosphate aminotransaminase 1 (GFAT1), the rate-limiting enzyme in the *O*-GlcNAcylation pathway [8], was significantly reduced at 19 h post-hCG but notably increased at 24 h post-hCG, though still below the 14 h level (Fig. 1H lane 3 and 1I). These enzymatic changes align with decreased *O*-GlcNAcylation signaling in aging oocytes, evidenced by reduced *O*-N-acetylglucosamine (*O*-GlcNAc) glycosylation intensity detected via Western blot (Fig. 1H lane 4 and 1I).

### Increased free Fe^2+^ and oxidative damage in aging oocytes

**Figure 2.**
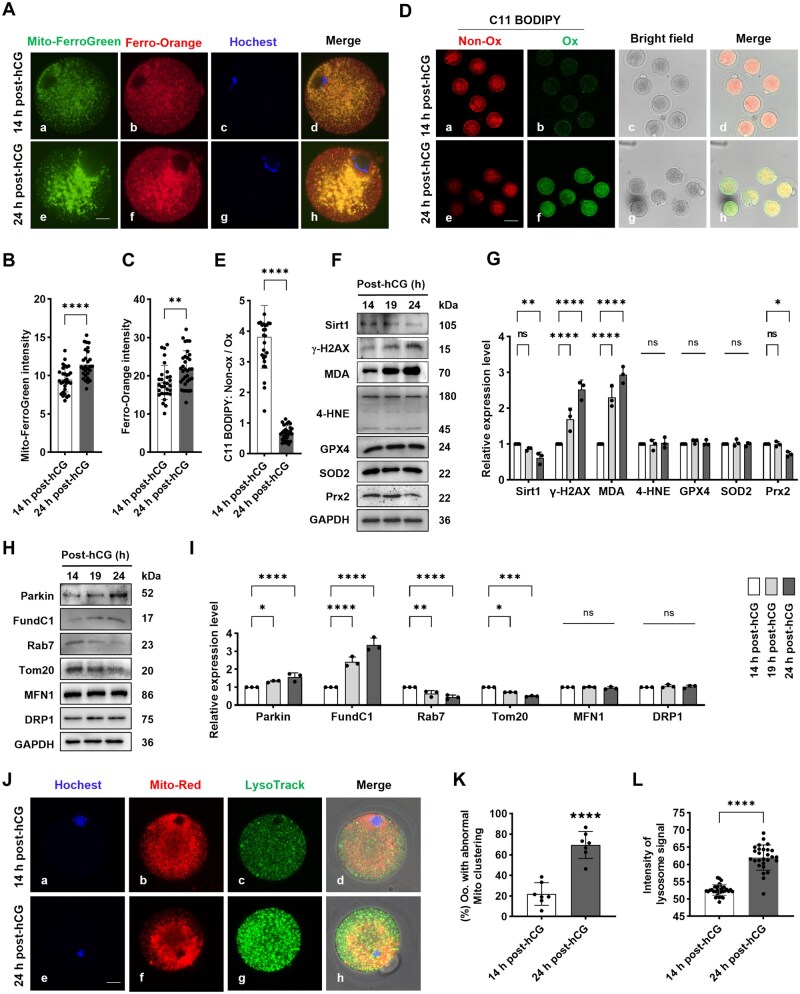
Increased intra-oocyte Fe^2+^ and oxidative stress damage during postovulatory aging. (A) Representative images of Fe^2+^ abundance and distribution in mouse oocytes. Oocytes were processed with live cell imaging using fluorescence probes Mito-FerroGreen and FerroOrange. Scale bar = 20 µm. (B, C) Statistical analysis of Fe^2+^ signal intensity on mitochondria and across cytosolic area in different groups. (D) Representative images of lipid peroxidation labeled by C11BODIPY581/591 fluorescent probe in mouse oocytes. Scale bar = 100 µm. (E) Statistical analysis about C11BODIPY signal intensity in mouse oocytes from different groups. (F) Representative immune blots of Sirt1, γ-H2AX, MDA, 4-HNE, GPX4, Prx2 and SOD2 in oocytes. (G) Statistical analysis about the gray scale intensity of Sirt1, γ-H2AX, MDA, 4-HNE, GPX4, Prx2 in oocytes from different groups. (H) Representative immune blots of Parkin, Rab7, FundC1, Tom20, MFN1 and DRP1 in mouse oocytes. (I) Statistical analysis about the gray scale intensity of Parkin, Rab7, FundC1, Tom20, MFN1 and DRP1 in oocytes from different groups. (J) Representative images about the distribution characteristics of mitochondria and lysosomes in mouse oocytes. Oocytes were processed with live cell imaging by using fluorescent probes MitoTracker (red) and LysoTracker (green), respectively. Scale bar = 20 µm. (K) Statistical analysis about the proportion of oocytes with abnormal mitochondria distribution in groups of 14 h and 24 h post-hCG. *n* = 38. (L) Statistical analysis about the signal intensity of lysosomes in oocytes at 14 h and 24 h after hCG administration. *n* = 36. All data were presented as the mean percentage (mean ± SEM) of at least three independent experiments. ns, *P* > 0.05, *P < 0.05, **P < 0.01, ***P < 0.001, ****P < 0.0001. Figure 2A–E and J–L were tested by *t*-test analysis, and Fig. 2F–I were tested by ordinary two-way ANOVA analysis.

To further investigate Fe^2+^ dynamics, we employed the fluorescent probes FerroOrange and Mito-FerroGreen, which specifically target cytoplasmic and mitochondrial Fe^2+^, respectively. At 14 h post-hCG, both probes revealed a moderate and evenly distributed Fe^2+^ signal throughout the cytosol in oocytes, with subtle granular enrichment near the presumptive spindle region. In contrast, by 24 h post-hCG, a significant increase in Fe^2+^ fluorescence was observed, now highly aggregated into large, distinct patches. Notably, the localization patterns of Fe^2+^ detected by both probes showed tight colocalization (Fig. 2A–C). These results indicate that iron homeostasis was disturbed in aging oocytes, the active Fe^2+^ ions were excessively released and mainly accumulated in mitochondria.

We next assessed lipid peroxidation in aging oocytes using the C11 BODIPY581/591 fluorescent probe. Consistent with expectations, lipid peroxidation levels were elevated, evidenced by a distinct spectral shift from red to green fluorescence in oocytes at 24 h post-hCG. Quantitatively, the red-to-green fluorescence ratio was significantly decreased (Fig. 2D and 2E). Western blot analysis further corroborated this finding, revealing a marked increase in malonaldehyde (MDA), the end product of lipid peroxidation, at 19 h and 24 h compared to 14 h post-hCG (Fig. 2F lane 3 and 2G). Furthermore, the level of γ-H2AX, a marker for double-stranded DNA breaks (DSBs), increased progressively, whereas the anti-aging factor Sirt1 significantly declined at 19 h and 24 h (Fig. 2F lane 1–2 and 2G). Concurrently, Prx2 levels decreased, while those of SOD2 and glutathione peroxidase 4 (GPX4) remained largely unchanged (Fig. 2F lane 5–7 and 2G). Notably, the cellular content of 4-HNE, a marker often associated with ferroptosis, did not exhibit significant alterations (Fig. 2F lane 4 and 2G).

Given that Fe^2+^ released from FHC degradation is primarily transported to mitochondria [9], we investigated mitochondrial responses to Fe^2+^ accumulation. Western blot analysis revealed an increasing trend in the protein levels of Parkin and FundC1, mediators of mitochondrial autophagy [10], in oocytes post-ovulation. Specifically, Parkin levels were significantly elevated at 24 h post-hCG compared to 14 h, while FundC1 was notably enhanced at 19 h and further increased at 24 h (Fig. 2H lane 1–2 and 2I). Conversely, the expression of Rab7, a negative regulator of mitochondrial autophagy [11], decreased significantly after 14 h post-hCG (Fig. 2H lane 3 and 2I). Additionally, the mitochondrial outer membrane marker protein Tom20 content was decreased (Fig. 2H lane 4 and 2I), whereas the levels of Mitofusin 1 (MFN1) and Dynamin 1-like (DRP1), regulators of mitochondrial fusion and fission [11], remained largely unchanged (Fig. 2H lane 5–6 and 2I). These data suggest that mitophagy is accelerated in aging oocytes. Consistent with this, MitoTracker (Red) staining at 24 h post-hCG revealed abnormal, patch-like mitochondrial aggregations (Fig. 2J and 2K), while LysoTracker (Green) labeling showed increased lysosomal signals throughout the cytoplasm (Fig. 2J and 2L). This implies that lysosomal degradation of ferritin releases Fe^2+^, potentially damaging mitochondria and consequently elevating mitophagy flux in aging oocytes.

**Figure 3.**
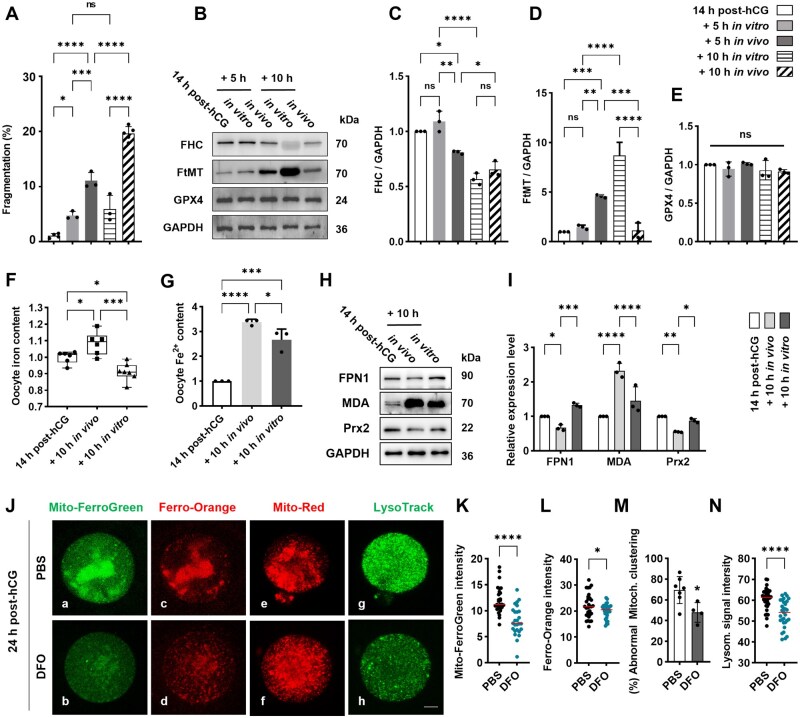
DFO rectified the abnormalities in Fe^2+^ and degenerative changes in oocytes cultured *in vitro* after ovulation. (A) Statistical analysis about the oocyte fragmentation in different groups. Optical microscopy analysis was conducted on oocytes from five groups: 14 h post-hCG, oocytes were collected from oviduct at 14 h post-hCG; +5 h *in vitro*, oocytes were collected from oviduct at 14 h post-hCG and cultured *in vitro* for 5 h in M2 medium; +10 h *in vitro*, oocytes were collected from oviduct at 14 h post-hCG and cultured *in vitro* for 10 h in M2 medium; +5 h *in vivo*, oocytes were collected from oviduct at 19 h post-hCG; +10 h *in vivo*, oocytes were collected from oviduct at 24 h post-hCG. (B) Representative immune blots of FHC, FtMT and GPX4 in oocytes from different groups. (C–E) Statistical analysis about the gray scale intensity of FHC, FtMT and GPX4 in oocytes from different groups. (F) The change of iron content in oocytes during postovulatory aging *in vitro* or *in vivo*. ICP–MS was conducted on oocytes from groups of 14 h post-hCG, +10 h *in vivo* and +10 h *in vitro*. (G) The change of ferrous ion level in aging oocytes. Colorimetric determination of ferrous ion was conducted on oocytes from groups of 14 h post-hCG, +10 h *in vivo* and +10 h *in vitro*. (H) Representative immune blots of FPN1, MDA and Prx2 in oocytes from groups of 14 h post-hCG, +10 h *in vivo* and +10 h *in vitro*. (I) Statistical analysis about the gray scale intensity of FPN1, MDA and Prx2 in oocytes from different groups. (J) Representative images show the distribution of Fe^2+^ in mitochondria (a, b) and across the cytosolic area (c, d), the morphologic features of mitochondria (e, f) and lysosomes (g, h) in mouse oocytes. Oocytes were processed with fluorescence probes Mito-FerroGreen (green), MitRed (red), FerroOrange (red) and LysoTrack (green), respectively. Scale bar = 20 µm. (K–N) Statistical analysis shows the difference in fluorescence signal intensity and distribution between PBS and DFO group. All data were presented as the mean percentage (mean ± SEM) of at least three independent experiments. ns, *P* > 0.05, *P < 0.05, **P < 0.01, ***P < 0.001, ****P < 0.0001. Figure 3A–G were tested by ordinary one-way ANOVA analysis, and Fig. 3H–I were tested by ordinary two-way ANOVA analysis. Figure 3J–N were tested by *t*-test A analysis.

Optical microscopy analysis revealed lower fragmentation rates in oocytes collected at 14 h post-hCG and cultured *in vitro* for an additional 5 h or 10 h in M2 medium, compared to their *in vivo* counterparts recovered from oviducts at 19 h and 24 h post-hCG, respectively. Furthermore, no significant differences in the fragmentation index were observed among the 14 h post-hCG group, the 14 h post-hCG + 5 h *in vitro* group, and the 14 h post-hCG + 10 h *in vitro* group (Fig. 3A). No significant differences in FHC or FtMT levels were observed between oocytes at 14 h post-hCG and those cultured *in vitro* for an additional 5 h (14-h-post-hCG + 5 h *in vitro*). In contrast, 10 h of *in vitro* culture (14-h-post-hCG + 10 h *in vitro*) resulted in decreased FHC and increased FtMT levels. This pattern differed from *in vivo* observations, where FHC expression was reduced at 19 h post-hCG and further lowered at 24 h post-hCG, while FtMT levels initially increased at 19 h post-hCG before decreasing significantly at 24 h post-hCG. Interestingly, the FtMT level peaked in the 14-h-post-hCG + 10 h *in vitro* group, surpassing levels in oocytes from both 19 h and 24 h post-hCG *in vivo* (Fig. 3B–D). ICP–MS/MS and colorimetric assays detected significantly lower absolute iron (Fig. 3F) and free Fe^2+^ (Fig. 3G) in oocytes from the 14-h-post-hCG + 10 h *in vitro* group versus the 24 h post-hCG *in vivo* group. Notably, FPN1 levels were significantly higher in the *in vitro*-aged oocytes (14-h-post-hCG + 10 h *in vitro*), indicating sustained iron excretion capacity (Fig. 3H lane 1 and 3I). This lower iron content correlated with significantly reduced MDA and elevated Prx2 levels in the *in vitro* group compared to the *in vivo* group (Fig. 3H lane 2–3 and 3I). In contrast, GPX4 levels remained unchanged across groups (Fig. 3B and 3E). Thus, the reduced iron load in *in vitro*-aged oocytes may account for the lower observed lipid peroxidation.

### DFO treatment mitigated alterations in ferritinophagy, Fe^2+^ levels, mitophagy, and *O* -GlcNAcylation within aging oocytes

We assessed if iron chelation via intraperitoneal deferoxamine (DFO) administration could mitigate intra-oocyte iron imbalance and lipid peroxidation. Fluorescence imaging showed that DFO significantly improved Fe^2+^ distribution and reduced its aggregation in 24 h post-hCG oocytes (Fig. 3J a–d ). Quantification confirmed reduced overall Fe^2+^ signal intensity and patch-like clustering, detected in mitochondria and the cytoplasm using Mito-FerroGreen and Ferro-Orange (Fig. 3K and 3L). Additionally, the frequency of oocytes with clustered mitochondria (visualized by Mito-Red) was lower in the DFO group than the PBS group (Fig. 3J e–f and 3M). Lysosomal activity, as indicated by LysoTrack signal intensity, was also markedly decreased in the DFO group (Fig. 3J g–h and 3N), implying less lysosome-driven ferritin breakdown and Fe^2+^ liberation.

**Figure 4.**
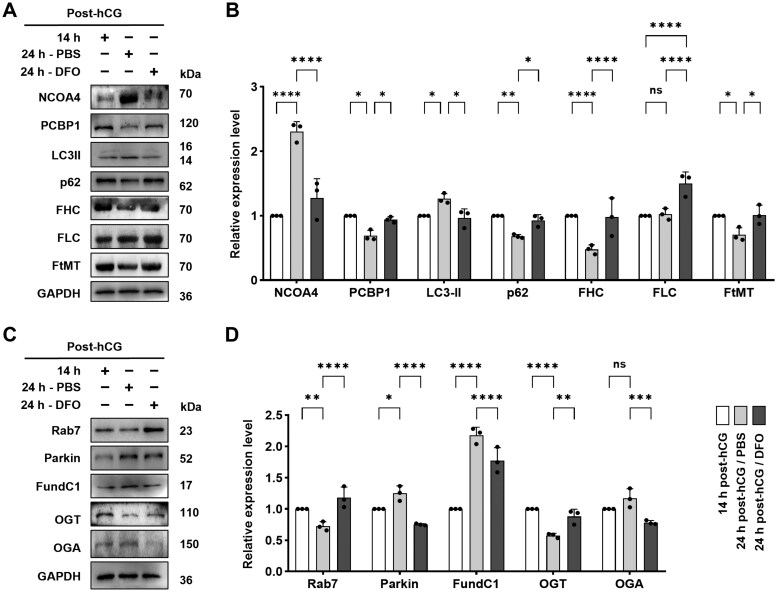
DFO rectified the changes in ferritinophagy, mitophagy and *O*-GlcNAcylation in aging oocytes. (A) Representative immune blots of NCOA4, PCBP1, LC3-II, p62, FHC, FLC and FtMT in mouse oocytes. (B) Statistical analysis about the blot gray intensity of NCOA4, PCBP1, LC3-II, p62, FHC, FLC and FtMT in groups of 14 h post-hCG, 24 h post-hCG/PBS and 24 h post-hCG/DFO. (C) Representative Western blots of Rab7, Parkin, FundC1, OGT and OGA proteins in mouse oocytes. (D) Statistical analysis about the blot gray intensity of Rab7, Parkin, FundC1, OGT and OGA in groups of 14 h post-hCG, 24 h post-hCG/PBS and 24 h post-hCG/DFO. All data were presented as the mean percentage (mean ± SEM) of at least three independent experiments. ns, *P* > 0.05, *P < 0.05, **P < 0.01, ***P < 0.001, ****P < 0.0001. Figure 4A–D were tested by ordinary two-way ANOVA analysis.

Western blot analysis showed that DFO treatment reduced NCOA4 and LC3II (Fig. 4A lane 1, 3 and 4B) and increased PCBP1 and p62 (Fig. 4A lane 2, 4 and 4B) compared to the PBS group, indicating suppression of excessive autophagy. This effect coincided with restored FHC, FLC and FtMT expression (Fig. 4A lane 5–7 and 4B) to levels akin to 14 h post-hCG. Furthermore, Rab7 levels were higher, but Parkin and FundC1 levels were significantly lower in the DFO group (Fig. 4C lane 1–3 and 4D), implying reduced mitophagy. Notably, DFO increased OGT and decreased OGA (Fig. 4C lane 4–5 and 4D) relative to PBS, levels nearing those at 14 h post-hCG. This change suggests enhanced *O*-GlcNAcylation, aligning with diminished ferritophagy in the DFO group.

### DFO ameliorated lipid peroxidation and apoptosis in aging oocytes

**Figure 5.**
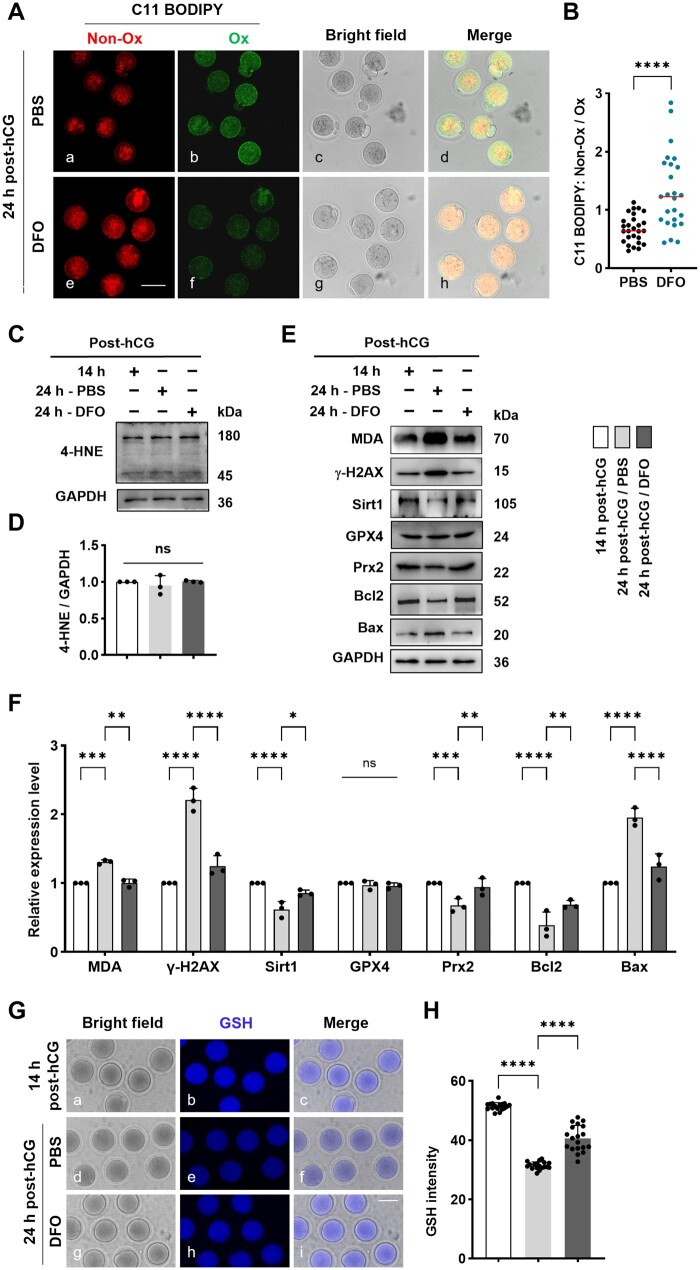
DFO ameliorated the lipid peroxidation damage and apoptosis in aging oocytes. (A) Representative images of lipid peroxidation labeled with C11BODIPY581/591 fluorescent probe in oocytes. Oocytes were processed with live cell imaging after labeled with C11BODIPY581/591 fluorescence probe, which labels membranes and fluoresces red in the intact state but shifts to green upon free radical-induced oxidation. Scale bar = 100 µm. (B) Statistical analysis of the intensity ratio of C11BODIPY Non-Ox to Ox signal between 24 h post-hCG/PBS group and 24 h post-hCG/DFO group. (C) Representative Western blot of 4-HNE level in oocytes. (D) Statistical analysis of blot gray intensity of 4-HNE in oocytes from groups of 14 h post-hCG, 24 h post-hCG/PBS and 24 h post-hCG/DFO. (E) Representative immune blots of MDA, γ-H2AX, Sirt1, GPX4, Prx2, Bcl2 and Bax in oocytes. (F) Statistical analysis about the blot gray intensity of MDA, γ-H2AX, Sirt1, GPX4, Prx2, Bcl2 and Bax in oocytes from groups of 14 h post-hCG, 24 h post-hCG/PBS and 24 h post-hCG/DFO. (G) Representative images of GSH in oocytes. Oocytes were incubated with 20 mM ThiolTracker Violet, glutathione detection reagent, and processed for image taking using an epifluorescence microscope with UV filters (370 nm, blue). Scale bar = 100 µm. (H) Statistical analysis of GSH level in groups of 14 h post-hCG, 24 h post-hCG/PBS and 24 h post-hCG/DFO. 72 oocytes were labeled. All data were presented as the mean percentage (mean ± SEM) of at least three independent experiments. ns, *P* > 0.05, *P < 0.05, **P < 0.01, ***P < 0.001, ****P < 0.0001. Figure 5A–D, 5G and 5H were tested by *t*-test, and Fig. 5E and 5F were tested by ordinary two-way ANOVA analysis.

Combined with live cell imaging, the ROS fluorescence probe DCFH-DA detected higher ROS signal in oocytes from PBS group than 14 h post-hCG group, but this increase was effectively pulled down in DFO group (Fig. S1A and S1B). At the same time, the lipid peroxidation fluorescent probe C11 BODIPY581/591 revealed spectrum transfer from red to green in oocytes from DFO group (Fig. 5A), the red to green ratio was significantly higher in DFO group than PBS group (Fig. 5B). Western blot showed that 4-HNE level was not changed (Fig. 5C and 5D), while MDA was significantly increased in PBS group, but this trend was obviously reversed in DFO group (Fig. 5E lane 1 and 5F). These findings suggest that intraperitoneal DFO administration effectively reduces ROS generation and lipid peroxide accumulation during oocyte aging. Consistently, DFO also corrected the changing trend in γ-H2AX (Fig. 5E lane 2 and 5F) and Sirt1 (Fig. 5E lane 3 and 5F), and improved the cellular antioxidant function, as demonstrated by the returned levels of Prx2, unexpectedly, DFO had no apparent effect on GPX4 expression (Fig. 5E lane 4–5 and 5F). In addition, DFO markedly restrained the downtrend of glutathione (GSH) level in oocytes from PBS group (Fig. 5G and 5H).

The decline in B cell leukemia/lymphoma 2 (Bcl2), an anti-apoptotic factor counteracting mitochondrial-mediated cell death [12], observed in oocytes at 24 h post-hCG was effectively reversed by DFO (Fig. 5E lane 6 and 5F), meanwhile the increase in Bax, a pro-apoptotic Bcl-2 family protein [12], associated with oocyte aging was also corrected by DFO administration (Fig. 5E lane 7 and 5F). These results collectively suggest that DFO alleviates oxidative stress and apoptosis in oocytes during post-ovulatory aging.

### DFO delayed the quality decline of oocytes in oviducts

**Figure 6.**
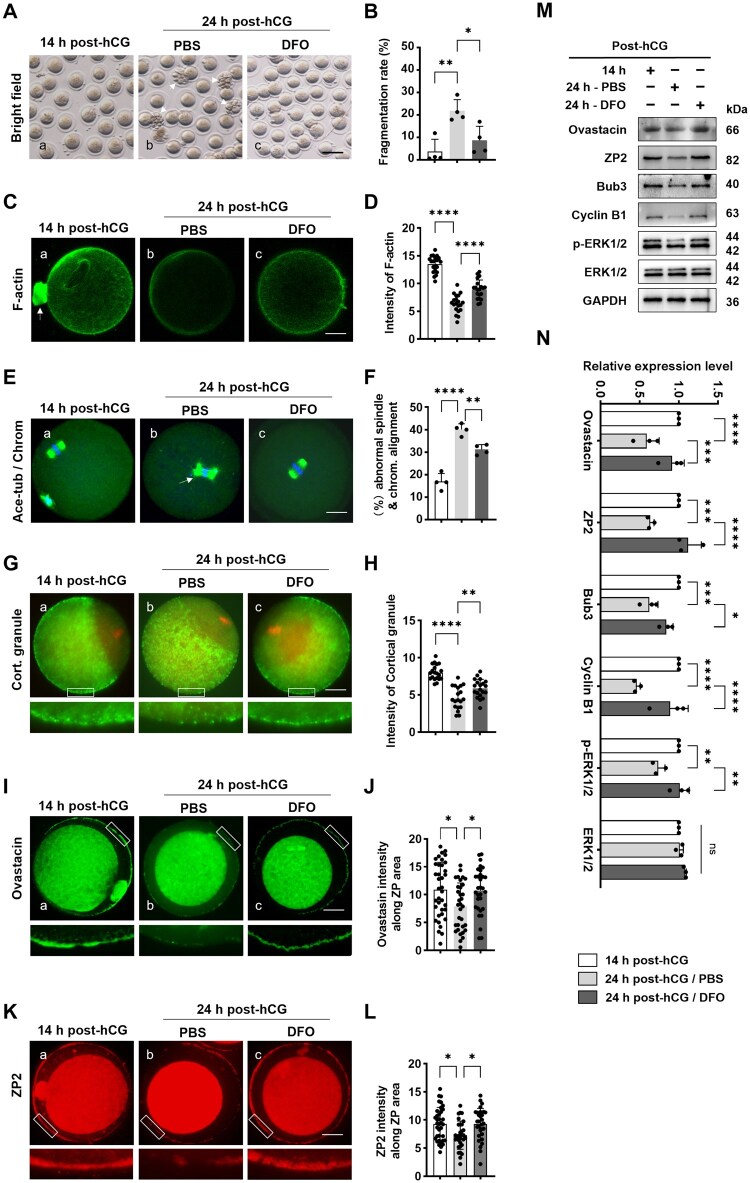
DFO rescued the quality of oocytes detained in oviducts after ovulation. (A) Representative photocomic images of oocytes from groups of 14 h post-hCG, 24 h post-hCG/PBS and 24 h post-hCG/DFO. The arrows indicate fragmented oocytes. Scale bar = 200 µm. (B) Statistical analysis about oocyte fragmentation in different groups. (C) Representative images of F-actin in oocytes. Oocytes were labeled with CellMask Green Actin Tracking stain and live cells were photographed using live-cell imaging system (Ex 503 nm/Em 512 nm, green). Scale bar = 20 µm. (D) Statistical analysis about signal intensity of F-actin in oocytes. (E) Representative images of spindle and chromosome morphology in oocytes. Spindle was immunolabeled with acetylated-α-tubulin (Ace-tubulin) in green, and chromosomes were labeled with DAPI in blue. The arrow in b indicates chromosomes scattered outside of the metaphase plate in aging oocytes. Scale bar = 20 µm. (F) Statistical analysis about oocytes with abnormal spindle and chromosomes in different groups. (G) Representative images of cortical granules (CG) in oocytes. Oocytes were fixed and stained with fluorescein isothiocyanate (FITC)-labelled Peanut Aggutinin (PNA), and imaged with laser confocal microscope. Scale bar = 20 µm. (H) Statistical analysis about signal intensity of CG in oocytes. (I) Representative images of Ovastacin in mouse oocytes. (J) Statistical analysis about signal intensity of Ovastacin in oocytes. (K) Representative images of ZP2 in mouse oocytes. (L) Statistical analysis about signal intensity of ZP2 in oocytes. Oocytes were fixed and immune-stained with antibodies to Ovastacin (green) and ZP2 (red), and imaged with confocal microscope. The region in the white frame was enlarged for detailed view. Scale bar = 20 µm. (M) Representative Western blot images of ZP2, Ovastacin, Bub3, CyclinB1, ERK1/2 and p-ERK1/2 in oocytes. (N) Quantitative analysis of protein level changes in oocytes. All data were presented as the mean percentage (mean ± SEM) of at least three independent experiments. ns, *P* > 0.05, *P < 0.05, **P < 0.01, ****P < 0.0001. Figure 6A–L were tested by ordinary one-way ANOVA analysis, and Fig. 6M and 6N were tested by ordinary two-way ANOVA analysis.

By quantitative analysis under the stereoscope, over 20% of the oocytes were fragmented in PBS group, these cells were also frequently featured with enlarged perivitelline space and degraded first polar body (Fig. 6A). In contrast, the proportion of such abnormal changes were significantly lowered in DFO group, near to that at 14 h post-hCG (Fig. 6B). As illustrated with CellMask Green Actin Tracking stain, F-actin was mainly distributed beneath the cytoplasm membrane and around the chromosomes in oocytes at 14 h post-hCG, however, in oocytes from PBS group, the cortical actin assembly was substantially decreased, and not arranged in a continuous and uniform strip beneath the cell membrane, as expected, this alteration was successfully rescued by DFO administration (Fig. 6C and 6D). As revealed by immunofluorescence, at 14 h post-hCG, the majority of oocytes contained bipolar barrel-shaped spindles, with neatly aligned chromosomes, however at 24 h post-hCG, nearly 40% oocytes were equipped with abnormal spindles, along with misaligned or scattered chromosomes, but such morphological abnormalities were considerably alleviated by DFO (Fig. 6E and 6F). Furthermore, the fluorescence intensity of cortical granules (CG) was significantly reduced in aging oocytes from PBS group, notably these CG were arranged in a discontinuous pattern, as if the particles had retreated into the cytoplasm area. In contrast, in oocytes from DFO-treated mice, the CG fluorescence intensity and its continuity was definitely improved, similar with that in oocytes at 14 h post-hCG (Fig. 6G and 6H).

Zona pellucida sperm-binding protein 2 (ZP2) and Ovastacin work together to ensure sperm-egg recognition, acrosome reaction and the prevention of polyspermy [13]. The immunofluorescence showed that ZP2 and Ovastacin were continuously and linearly distributed along the zona pellucida in freshly ovulated oocytes (14 h post-hCG), in contrast, the signals of these two molecules were discontinuous in aging oocytes from PBS group (Fig. 6I–L), actually their protein content were markedly reduced, as evidenced by Western blot and relative quantification analysis (Fig. 6M lane 1-2 and 6N). Undoubtedly, this aging-related change was dramatically reversed by DFO, in terms of both the subcellular distribution and the protein expression level (Fig. 6I–N).

We found that a considerable proportion of aging oocytes had entered the second anaphase of meiosis (data not showed). In logical consistence with this, the protein levels of Bub3 mitotic checkpoint protein (Bub3), Cyclin B1 and phosphorylated ERK1/2 (p-ERK) were all significantly decreased in oocytes from PBS group, which explains, at least partially, why the aging oocytes are more easily activated [14, 15]. In the group of DFO treatment, these proteins were definitely rescued and nearly equal to that in fresh oocytes from 14 h post-hCG group (Fig. 6M lane 3–5 and 6N). In addition, there was no obvious difference in the total level of ERK1/2 among these three groups (Fig. 6M lane 6 and 6N).

### DFO improved the fertilization competence and early embryo development

**Figure 7.**
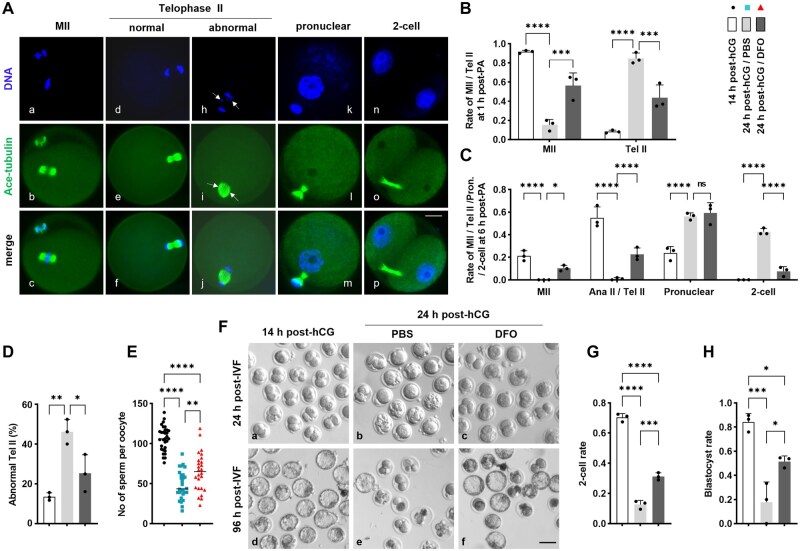
DFO improved the early development of aging oocytes after parcinogenetic activation and fertilization. (A) Representative images of developmental stages at MII, Tel II, pronuclear and 2-cell. Oocytes were cultured for 1 h or 6 h after parthenogenetically activated with 7% ethanol, then immune-stained with ace-tubulin (green), with the chromosomes labeled with DAPI in blue. Arrows in i indicate asymmetrical spindle configuration; Arrows in h indicate lagging chromosomes. Scale bar = 20 µm. (B) Quantitative analysis of oocytes at MII or Tel II at 1h after parthenogenesis activation in groups of 14 h post-hCG, 24 h post-hCG/PBS and 24 h post-hCG/DFO. (C) Quantitative analysis of developmental stages at MII, Tel II, pronuclear and 2-cell at 6 h after parthenogenetic activation in different groups. (D) The proportion of Tel II with abnormal spindle and chromosome separation in groups of 14 h post-hCG, 24 h post-hCG/PBS and 24 h post-hCG/DFO. (E) Quantitative analysis of the number of sperm bound to per oocytes during *in vitro* fertilization. After incubated with sperm for 4 h, the oocytes with bound or incorporated sperm were fixed and labeled with DAPI, the sperm heads on each oocyte were counted under fluorescence microscope. (F) Representative images of embryos at 24 h and 96 h after *in vitro* fertilization. IVF, *in vitro* fertilization. (G–H) Statistical analysis of 2-cell and blastocyst embryos in groups of 14 h post-hCG, 24 h post-hCG/PBS and 24 h post-hCG/DFO. All data were presented as the mean percentage (mean ± SEM) of at least three independent experiments. *P < 0.05, **P < 0.01, ***P < 0.001, ****P < 0.0001. Figure 7B and 7C were tested by ordinary two-way ANOVA analysis, and Fig. 7D, 7G and 7H were tested by ordinary one-way ANOVA analysis.

We continued to explore the developmental features of early embryos after parthenogenetic activation (PA) and *in vitro* fertilization in groups of 14 h post-hCG, 24 h post-hCG/PBS and 24 h post-hCG/DFO. The developmental stage was determined by immunostaining of microtubules and DNA. The majority of oocytes in 14 h post-hCG group remained at the MII stage at 1 h after PA with 7% ethanol, meanwhile there about 85.66% oocytes in PBS group had entered telophase II (Tel II), notably, this raising proportion was significantly lowered in DFO group, that is about 56.33% oocytes remained at MII stage, and only about 43.67% transferred to Tel II stage (Fig. 7A and 7B). At 6 h after PA, in the group of 14 h post-hCG, about 21.33% oocytes were still at MII, 55.00% at Tel II and 23.67% at pronuclear stage, while in PBS group, there were no cells at MII and only 1.00% at Tel II, but about 56.33% were at pronuclear and 42.67% had reached to 2-cell stage. In DFO group, there were about 10.33% oocytes remained at MII, 22.67% at Tel II, 59.33% at pronuclear stage and only 7.67% at 2-cell stage (Fig.7A and 7C). Furthermore, Tel II oocytes in the PBS group often displayed disordered spindles and asymmetric chromosome segregation, with an incidence significantly higher than that in the 14 h post-hCG group. This increase in abnormalities was markedly mitigated by DFO treatment (Fig. 7A and 7D). These data indicate that the aging oocytes were activated more easily and developed more faster to 2-cell stage. Notably, this accelerated developmental trend could be reversed by the pre-application of DFO.

In consistence with the abnormal dynamics of CGs, ZP2 and Ovastacin, the number of sperms bound to each oocyte during *in vitro* fertilization was significantly lower in PBS group than that in 14 h post-hCG group, however, this number was reversed again in DFO group (Fig. 7E), suggesting DFO pre-application could rescue oocyte ability of binding sperm. In addition, the aging oocytes exhibited poor development viability to 2-cell and blastocyst stage when checked at 24 h and 96 h after *in vitro* fertilization, and as expected, DFO administration could improve these two developmental indicators (Fig. 7F–H).

### HO-1 inhibition decreased intra-oocyte iron and degenerative changes with time post-ovulation

To determine if oviductal HO-1 overexpression relates to iron abnormalities in aging oocytes, we inhibited HO-1 using intraperitoneal ZnPP injection at 14 h post-hCG. Oocytes were then collected from oviducts at 24 h post-hCG for subsequent analysis. As confirmed by colorimetric assay, the intra-oocyte ferrous iron level was dramatically reduced in 24 h post-hCG/ZnPP group (ZnPP group) compared to 24 h post-hCG/vehicle group (Vehicle group) (Fig. S1C). Simultaneously, oocyte fragmentation rate and intracellular ROS level (DCFH-DA signal), were significantly decreased in ZnPP group (Fig. S1D–G). The level of MDA was decreased while that of Prx2 was increased in oocytes from ZnPP group than the vehicle control (Fig. S1H–J).

## Discussion

**Figure 8.**
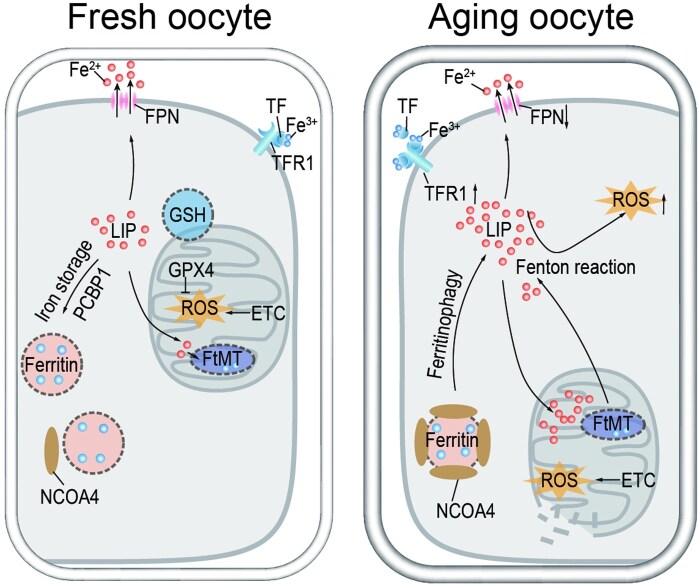
Graphical summary. In oocytes during postovulatory aging, the levels of ferritinophagy and cytosolic Fe^2+^ are increased dramatically, which further cause mitophagy enhancement and lipid peroxidation injury, weakening oocyte quality and developmental potential.

This study shows that higher iron levels during postovulatory aging increase ferritinophagy, mitophagy, and lipid peroxidation injury in mouse oocytes. *In vivo* iron chelation alleviates iron dysregulation and peroxide buildup post-ovulation (Fig. 8), thereby boosting fertilization rates and early embryo development. These results imply that iron metabolic imbalance is a significant contributor to ROS production and oocyte aging after ovulation in mammals, revealing a novel link between oocyte aging and iron homeostasis that could inform new treatments for reproductive issues.

Iron overload is firmly associated with a range of age-related degenerative disorders, particularly neurodegenerative diseases such as Alzheimer’s, Parkinson’s, and multiple sclerosis [16–18]. Importantly, iron chelation therapy has demonstrated the ability to significantly reduce the neural degenerative changes characteristic of these conditions [11, 19]. Furthermore, iron overload is also recognized as a contributing factor in female reproductive system pathologies, including premature ovarian failure, endometriosis, preeclampsia, and spontaneous abortion, particularly in older women [20, 21]. Our findings demonstrate that disruption of iron homeostasis occurs in oocytes during postovulatory aging, marked by elevated iron levels, particularly cytoplasmic Fe^2+^. We propose that this is, at least in part, driven by high HO-1 expression in the oviduct, which increases Fe^2+^ production from heme [5]. Notably, oviductal HO-1 is low at 14 h post-hCG, the optimal fertilization window for oocytes. As HO-1 levels rise over time, enhanced heme breakdown elevates oviductal Fe^2+^, establishing an iron-rich microenvironment that promotes oocyte iron uptake.

It is known that mouse oocytes usually degenerate 12 h post-ovulation in the oviduct, but survive longer *in vitro* [22]. Here, we found higher iron, especially ferrous iron, in oocytes left in the oviduct compared to those cultured *in vitro* for the same time. This difference might be due to higher HO-1 in the oviduct tissue, possibly explaining why oocytes in the oviduct degenerate faster. Previous studies established that microglial HO-1 overexpression is linked to neurotoxic iron accumulation in aging mice, and that HO-1 inhibition can ameliorate iron status and related degenerative changes [23, 24]. Consistent with these findings, our work confirms that HO-1 inhibition alleviates iron accumulation and oxidative stress damage *in vivo* during oocyte aging. We propose that this beneficial effect stems from reduced heme-derived iron generation, which alleviates iron overload and restores redox balance within the oviduct environment.

Previous studies have demonstrated that elevated iron levels diminish protein *O*-GlcNAcylation in cultured 3T3-L1 adipocytes and in mice fed high-iron diets [25]. This suggests that the reduced *O*-GlcNAcylation observed in aging oocytes may also be attributed to elevated iron levels. The de-*O*-GlcNAcylation of FHC promotes its NCOA4-mediated degradation via autophagy, thereby releasing Fe^2+^ into the cytoplasm [8]. While the decline in IRP2 levels in aging oocytes might alleviate translational suppression of FHC [26], newly synthesized FHC may be insufficient to compensate for protein loss due to lysosomal degradation, particularly given the cessation of new mRNA production post-ovulation. Consequently, as oocytes reside longer in the fallopian tubes, intra-oocyte ferritinophagy becomes increasingly active, leading to significant Fe^2+^ release from ferritin degradation. Ferritinophagy typically leads to the accumulation of labile iron near mitochondria, which act as a temporary reservoir for this metal [8, 9]. The rising Fe^2+^ levels may eventually surpass mitochondrial buffering capacity, causing lipid peroxidation damage to mitochondrial membranes [8, 11, 27].

Taken together, the data from this study point to iron metabolic dysregulation as the central mechanism leading to excessive ROS and oocyte degeneration during postovulatory aging. By uncovering this previously unknown association between oocyte aging and iron homeostasis, our work suggests new possibilities for therapeutic interventions aimed at improving reproductive health.

### Research limitations

A notable limitation of the current study is that it did not examine the expression changes of ACSL4 and other ferroptosis-related markers in mouse oocytes during postovulatory aging. Investigating these alterations could offer crucial insights into the underlying mechanisms driving oocyte aging.

## Methods

### Research ethics

All the animal experiments were approved by the Animal Care and Use Committee of Capital Medical University with an approval number AEEI-2020-151, following the Administration Regulations on Laboratory Animals of Beijing Municipality. Female CB6F1 mice (F1-hybrid of C57BL/6 ♂ × BALB/C ♀), aged six to eight weeks, were bred in a specific pathogen-free environment with a 12 h light–dark cycle (lights on at 8:00 am and off at 8:00 pm daily) and free access to food and drinking water under constant conditions of temperature (25°C) and humidity (50%–60%). The mice were euthanized with CO_2_ just before sampling.

### Oocytes and oviducts collection

Mice were injected with 10 IU of pregnant mare serum gonadotropin (PMSG, Ning Bo Second Hormone Factory), followed by an injection of 10 IU of human chorionic gonadotrophin (hCG, Ning Bo Second Hormone Factory) after 48 h. At 14 h, 19 h, and 24 h after hCG injection, the cumulus-oocyte complexes (COCs) were harvested from the oviducts. Following the gentle pipetting up and down in Minimal Essential Medium (MEM) supplemented with 0.1% hyaluronidase (H3506, Sigma) to remove the cumulus cells, oocytes at metaphase II (MII) were harvested for subsequent analysis.

Along the scheduling of PMSG-hCG superovulation, the entire oviducts were sequentially collected at 0 h and 48 h post-PMSG, 14 h, 19 h and 24 h post-hCG, and immediately stored at −80°C for Western blot analysis.

### Parthenogenetic activation and *in vitro* fertilization

The parthenogenetic activation medium was 7% ethanol in MEM with 3 mg/mL crystallized bovine serum albumin (BSA, Sigma-Aldrich, A1933). The ovulated oocytes (metaphase II, MII) were first incubated in the activation medium for 5 min, then thoroughly washed, and cultured in fresh MEM/BSA for additional 1 h and 6 h to assess anaphase II progression and pronucleus formation.

For *in vitro* fertilization, the epididymides from 4-month-old CB6F1 males were minced in 900 µL MEM/BSA under oil, allowing the sperm swim for 12 min. Then 6 µL sperm solution was added into 500 µL MEM/BSA drop containing MII oocytes under oil for fertilization. At 4 h post-fertilization, the presumptive zygotes were washed and transferred to fresh 80 µL MEM/BSA drops under oil. Two-cell embryos at 24 h were transferred to KSOM media (Nanjing Aibei Biotechnology, LOT5015A) for development to the blastocyst stage. All cultures were conducted in an atmosphere of 5% CO_2_, 5% O_2_ and 90% N_2_ at 37°C.

### Determination of intra-oocyte iron content with ICP–MS/MS

Each sample contained 40 oocytes, which were lysed in 1 mL ultra-pure water and frozen at −80°C for storage. As determination began, the sample was added with 2 mL nitric acid and stood for 2 h, then heated in a water bath at 80°C for additional 2 h. After cooled to room temperature, the solution was stinted to 5 mL with ultra-pure water and injected to ICP–MS instrument (8800, Agilent) for iron content determination. The instrument parameters were set following the operational instructions, a standard curve was constructed while a regression equation was calculated accordingly.

### Colorimetric determination of intra-oocyte ferrous iron

The intra-oocyte ferrous iron level was determined by using the Cell Ferrous Iron Colorimetric Assay Kit (E-BC-K881-M, Elabscience). 320 oocytes were homogenized for 10 min in 200 µL lysis buffer using ultrasonic crushing on ice. The lysate was then centrifuged at 15,000× *g* for 10 min to separate the supernatant. Then 80 µL supernatant was incubated with either the iron probe or the control reagent for 10 min at 37°C. The absorbance was measured at 593 nm using a Multiscan FC plate reader and analyzed with Skanlt Multiscan FC software (Thermo Scientific). A standard curve of cellular ferrous iron content was used to quantify the ferrous iron levels in oocytes. The relative ferrous iron level was determined as the difference in absorbance between the experimental and control groups. The determinations were normalized to the total content.

### Drug treatment *in vivo*

#### Desferrioxamine (DFO) administration

DFO (HY-B0988, MCE) was properly dissolved in phosphate-buffered saline (PBS) and injected intraperitoneally into mice at 100 mg/kg each time. The control mice were injected with same volume of PBS. According to the injecting treatment in mice, oocytes were divided into three groups: (i) 14 h post-hCG, oocytes were obtained from mice at 14 h post-hCG without further treatment; (ii) 24 h post-hCG/PBS, oocytes were harvested at 24 h post-hCG from mice injected with PBS at 0 h and 14 h post-hCG; (iii) 24 h post-hCG/DFO, oocytes were collected at 24 h post-hCG from mice injected with DFO solution at 0 h and 14 h post-hCG.

#### Zn (II)-protoporphyrin IX (ZnPP) treatment

ZnPP (TargetMol, T13396) was dissolved in the vehicle solution (10% DMSO + 40% PEG2000 + 5% Tween-80 + 45% saline) and intraperitoneally injected into mice at 10 mg/kg each time. The control mice were injected with same volume of vehicle solution. According to the drug administration in mice, oocytes were classified into three groups: (i) 14 h post-hCG, oocytes were obtained from mice at 14 h post-hCG without further treatment; (ii) 24 h post-hCG/vehicle, oocytes were collected at 24 h post-hCG from mice injected with vehicle at 14 h post-hCG; (iii) 24 h post-hCG/ZnPP, oocytes were at 24 h post-hCG from mice injected with ZnPP solution at 14 h post-hCG.

### Immunofluorescence

After proper treatments, the oocytes were fixed in 1% paraformaldehyde (PFA) in PEM buffer (100 mM Pipes, 1 mM MgCl_2_, and 1 mM EGTA, pH 6.9) with 0.5% Triton X-100 for 45 min at room temperature. After washed in PBST (phosphate-buffered saline with 0.02% Triton X-100), the oocytes were blocked in PBS containing 10% normal goat serum and 1% BSA, and then incubated overnight at 4°C with diluted antibodies, as listed in Table S1. The oocytes were thoroughly washed three times in PBST and labeled with appropriate second antibodies for 1 h at room temperature under dark conditions. Following careful washing steps, the oocytes were mounted on slides using an antifade mounting medium containing DAPI (H-1200, Vector Laboratories) and examined using a fluorescent ZEISS microscope. Finally, the images were harvested by using a laser-scanning confocal microscope (Zeiss Axio Imager A2), and the fluorescence signal intensity was quantified densitometrically using ImageJ software.

### Western blot

80 oocytes were boiled for 7–10 min in Laemmli sample buffer (1610737, Bio-Rad) containing protease inhibitor cocktail (P2714, Sigma). The proteins were separated on a 10% SDS-PAGE gel and transferred onto PVDF membranes (IPVH00010, Millipore) using a constant current of 250 mA for 1.5 h. After that, the membranes were blocked with 5% fat-free milk in TBST (Tris-buffered saline with 0.1% Tween-20) at room temperature for 1 h before incubated overnight at 4°C with diluted primary antibodies in Table S1. After washing three times in TBST, 20 min each time, the membranes were incubated in horseradish peroxidase-conjugated secondary antibodies for 1 h at room temperature. The bands were visualized with enhanced chemiluminescence system (E412-01, Vazyme) and further processed for semiquantitative gray scale analysis using Image J software.

### Cellular and mitochondrial Fe^2+^ assay

The oocytes were processed with live cell imaging after labeled with fluorescent probes FerroOrange (F374, DojinDo) and Mito-FerroGreen (M489, DojinDo). During the oocyte incubation with the probes, Ferric iron (Fe^3+^) ions are dissociated from Ferritin and reduced to their divalent form (Fe^2+^), which then react with the probe to generate stable-colored complexes. Before use, Mito-FerroGreen and FerroOrange were respectively dissolved in DMSO at same concentration of 1 mmol/L as stock solution. The two stocks were simultaneously diluted in HBSS to make a working solution containing 5 μmol/L Mito-FerroGreen and 1 μmol/L FerroOrange. Denuded oocytes were incubated in the working solution for 30 min and then processed with live cell imaging on LSM 880 with Airyscan confocal laser scanning microscopes (Zeiss), under identical conditions for the control and experimental groups.

### Lipid peroxidation detection by C11-BODIPY581/591

The lipid peroxidation was assayed using the sensor C11-BODIPY581/591 (thermo. D3861, Invitrogen), which employs a fluorescent fatty acid analogue with inherent fluorescence properties. Following oxidation by free radicals, the fluorescence of this probe experiences a spectral transition from red to green, corresponding to a shift in the emission wavelength from 591 nm (in the reduced state) to 510 nm (in the oxidized state). The excitation wavelengths for these forms are 581 and 500 nm, respectively. This color change allows for the visualization of the oxidation process using confocal microscopy. The denuded oocytes were incubated in MEM with 10 μM C11-BDDIPY581/591 for 30 min at 37°C in 5% CO_2_. After thoroughly washed in fresh MEM, the cells were transferred to M2 medium (M7167, sigma) and processed with live cell imaging as mentioned above, and the analysis of fluorescence intensities using ImageJ software.

### Detection of cellular ROS

The denuded oocytes were labeled with 10 μM dichlorofluorescein diacetate green (DCFH-DA) (S0033, Beyotime) in MEM for 30 min at 37°C. After carefully washed and settled in fresh M2 medium, the oocytes were observed with a confocal microscope, as mentioned above. The fluorescence signal intensity was measured using ImageJ software.

### Mito-Tracker deep red and Lyso-Tracker green staining

The Mito-Tracker Deep Red FM powder (C1032, Beyotime) was dissolved in DMSO to achieve a stock solution at concentration of 200 μM. Before use, the stock was further diluted in M2 medium, resulting in a final concentration of 200 nM. The solution of Lyso Tracker Green (C1047S, Beyotime) was directly diluted in M2 medium to obtain a working concentration of 75 nM. The denuded MII oocytes were incubated in M2 medium with both working concentrations for 30 min in 5% CO_2_. After rinsed three times with fresh M2, oocytes were processed for direct observation and image acquisition with confocal microscopy as mentioned above.

### Green actin tracking staining

Oocytes were incubated in M2 medium containing 200 nM CellMask^TM^ Green actin tracking stain (A57243, Invitrogen) and 1 µg/mL Hoechst 33342 (4082, Cell Signaling Technology) for 30 min. After washed 6 times in fresh M2 medium, the oocytes were analyzed under confocal microscopy, as noted above.

### Cortical granules (CG) distribution assay

Oocytes were fixed in 4% PFA with 0.5% Triton X-100 in PEM buffer (*w*/*v*) for 45 min at room temperature. The cells were washed in PBST, and then incubated in 100 µg/mL FITC-Peanut Agglutinin (FITC-PNA, YJ70000852, Shanghai Yiji Chemical) and 1 µg/mL Hoechst for 30 min at room temperature in a dark chamber. After washed carefully, the oocytes were mounted on slides using an antifade mounting medium containing DAPI and examined using a fluorescent ZEISS microscope. The acquired images were analyzed to quantify fluorescence intensity densitometrically with ImageJ software.

### Statistical analysis

The data in Figs. 1B, 1C, 3A, 3C–G, 4D, 5D, 5H, 6B, 6D, 6F, 6H, 6J, 6L, 7D, 7E, 7G, 7H and Fig. S1B, S1C, S1E, S1G, S1I, S1J were analyzed using a one-way ANOVA test. Normality and lognormality tests were performed on all the data using GraphPad Prism software with Shapiro-Wilk or Kolmogorov-Smirnov *P* values > 0.1. Variance was tested using Brown-Forsythe test or Bartlett’s test with adjusted *P* values < 0.1 using the Welch method. A *t*-test was used to analyze the data in Figs. 2B, 2C, 2E, 2K, 2L, 3B–E and 5B after normality and lognormality tests showed Shapiro-Wilk or Kolmogorov-Smirnov *P* values > 0.1. All variances for these datasets were compared using an *F*-test with adjusted *P* values < 0.1 employing the Welch method. Two-way ANOVA test was employed to analyze the data in Figs. 1E, 1G, 1I, 2G, 2I, 3I, 4B, 4F, 5F, 6N, 7B and 7C. Normality tests conducted on all these datasets indicated Shapiro-Wilk *P* values > 0.1, and all data exhibited normal distribution and homogeneity of variance.

## Supplementary Material

lnaf032_Supplementary_Data

## Data Availability

The data that support the findings of this study are available from the corresponding author upon reasonable request.
