# Supplementary material for: Iron metabolism disorder promotes postovulatory oocyte aging by inducing oxidative stress damage
Source: Life Med. 2025 Sep 30;4(6):lnaf032. doi: 10.1093/lifemedi/lnaf032 (PMC12732666; doi:10.1093/lifemedi/lnaf032)
Supplement: lnaf032_Supplementary_Data [file lnaf032_supplementary_data.zip › SI.docx]

**Iron metabolism disorder promotes postovulatory oocyte aging by inducing oxidative stress damage**

Ziqi Huang^1^, Bicheng Wang^1^, Ying Tian^1^, Xiangning Xu^1^, Jiaqi Zhang^1^, Shuo Lou^1^, Jingyi Kang^1^, Ningning Zhang^1^, Ke Song^2^, Jingyu Li^2^, Jing Weng^1^, Yuanjing Liang^1^, Xiaokui Yang^2,*^, Wei Ma^1,*^

^1^Department of Histology and Embryology, School of Basic Medical Sciences, Capital Medical University, Beijing 100069, China

^2^Department of Human Reproductive Medicine, Beijing Obstetrics and Gynecology Hospital, Capital Medical University, Beijing 100020, China

^*^Correspondence: mawei1026@ccmu.edu.cn (W.M.), yangxiaokui@ccmu.edu.cn (X.Y.)

Figure S1. Decreased levels of intracellular iron and degenerative changes in aging oocytes from mice injected with DFO or ZnPP.

(A) Representative images of intracellular ROS labeled with DCFH-DA fluorescent probe in oocytes from groups of 14 h post-hCG, 24 h post-hCG/PBS and 24 h post-hCG/DFO. Oocytes were processed with live cell imaging after labeled with DCFH-DA probe (green). Scale bar = 100 μm. (B) Statistical analysis of the intensity of DCFH-DA signal among 14 h post-hCG, 24 h post-hCG/PBS group and 24 h post-hCG/DFO group. (C) Statistical analysis of ferrous ion level in aging oocytes. Colorimetric assay of ferrous ion was conducted on oocytes from groups of 14 h post-hCG, 24 h post-hCG/Vehicle and 24 h post-hCG/ZnPP. (D) Representative photocomic images of fragmentated oocytes from groups of 14 h post-hCG, 24 h post-hCG/Vehicle and 24 h post-hCG/ZnPP. The arrows indicate fragmented oocytes. Scale bar = 200 μm. (E) Statistical analysis about the oocyte fragmentation in different groups. (F) Representative images of intra-oocyte ROS labeled with DCFH-DA fluorescent probe in groups of 14 h post-hCG, 24 h post-hCG/Vehicle and 24 h post-hCG/ZnPP. Scale bar = 100 μm. (G) Statistical analysis of DCFH-DA intensity among groups of 14 h post-hCG, 24 h post-hCG/Vehicle and 24 h post-hCG/ZnPP. (H) Representative Western blot images of MDA and Prx2 in oocytes from different groups. (I, J) Quantitative analysis of the changes of MDA and Prx2 in oocytes. All data were presented as the mean percentage (mean ± SEM) of at least three independent experiments. **P* < 0.05, ***P* < 0.01, ****P* < 0.001, *****P* < 0.0001 by ordinary one-way ANOVA analysis.
