# Supplementary figures and images for: Iron metabolism disorder promotes postovulatory oocyte aging by inducing oxidative stress damage
Source: Life Med. 2025 Sep 30;4(6):lnaf032. doi: 10.1093/lifemedi/lnaf032 (PMC12732666; doi:10.1093/lifemedi/lnaf032)

Supplementary Figure 1

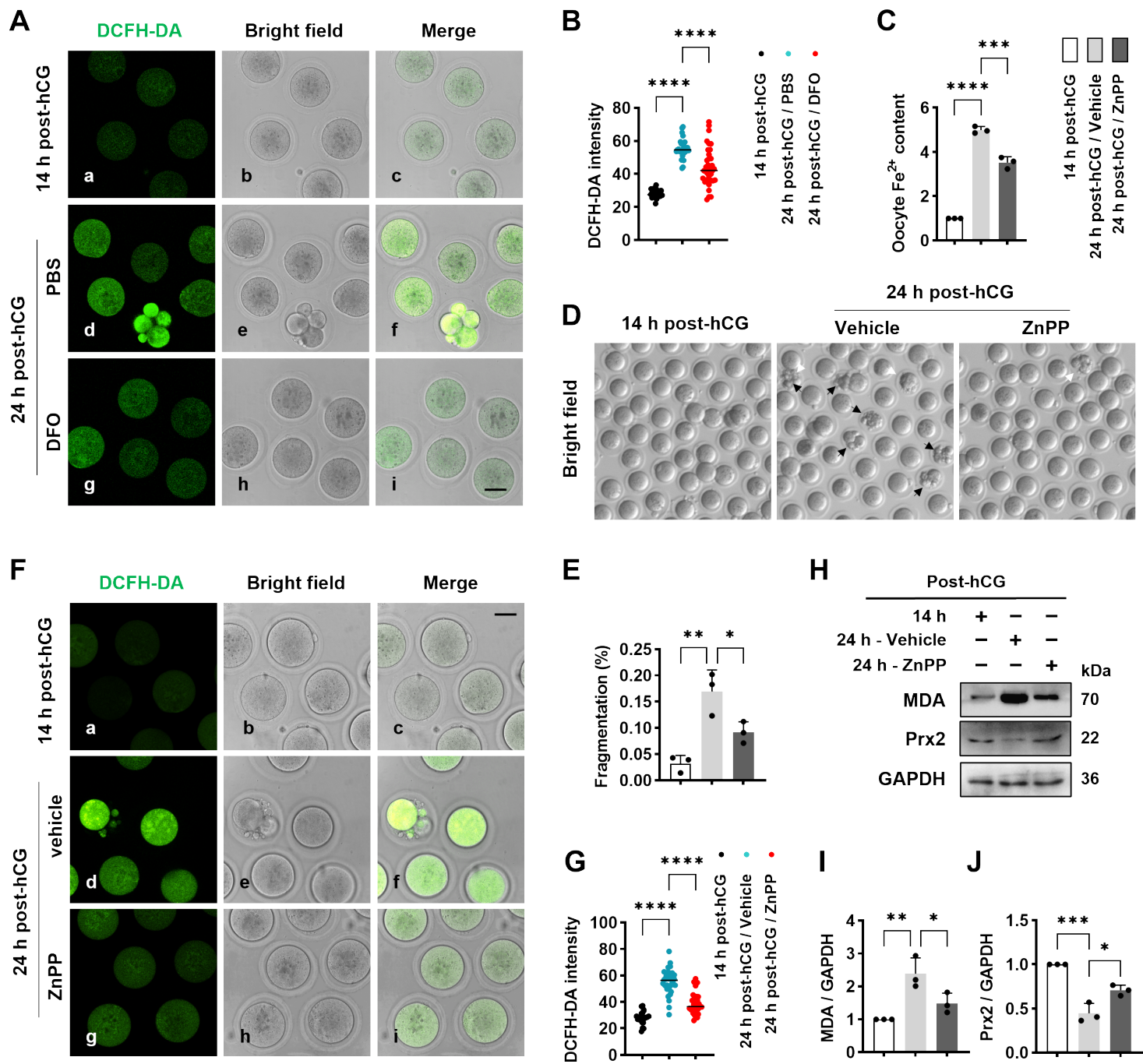

Supplement: lnaf032_Supplementary_Data [file lnaf032_supplementary_data.zip › Figure S1_PE.pdf]
